# Survey on opinions and usage patterns for the ResearchGate platform

**DOI:** 10.1371/journal.pone.0204945

**Published:** 2018-10-05

**Authors:** Andreas Meier, Dirk Tunger

**Affiliations:** Central Library, Forschungszentrum Jülich, Jülich, Germany; Charles Sturt University, AUSTRALIA

## Abstract

Based on a survey, the following study investigates opinions and also usage patterns relating to the ResearchGate social networking site for scientists and researchers. The survey consisted of 19 questions and was conducted online with 695 scientists from the disciplines of physics, biology, medicine, and neuroscience. Amongst other issues, the research questions concerned how much time and effort the interviewees expended on ResearchGate, what added value they perceived in using the site, the extent to which social aspects influence use, how participants planned to use the platform in future, and what role ResearchGate’s own metric, the RG score, played for the scientists. In addition, we discuss which of the factors of age, sex, origin, and scientific discipline have a decisive influence on the responses of the interviewees and which are of no statistical significance The results clearly show that the origin of the participants is frequently decisive, but that the remaining factors also have a considerable influence on the responses for more than 25% of the questions.

## Introduction

The wide range of possibilities offered by Science 2.0 means that scientists have many opportunities of managing and discussing their scientific output by managing bibliographic data, releasing their own publications online, or exchanging ideas with other scientists in social networks. For this purpose, researchers can make use of a wide range of diverse portals such as Academia.edu, ResearchGate, Mendeley, Bibsonomy, CiteULike and Zotero [1,2]. With more than 12 million users, ResearchGate is regarded as the most popular social network for scientists [3]. The network has its own impact indicator, the RG score, which is a type of unique selling point with respect to composite indicators among the social platforms. This score combines several dimensions including the influence of the user’s scientific publications and also their social activities on ResearchGate [4]. These involve asking and answering questions which can then be evaluated by other users [5]. Although the composition of the RG score in the form of a percentage distribution is transparent, the algorithm used for the calculation remains confidential [4]. This conflicts with the "Leiden Manifesto for research metrics" [6], since the calculation of the RG score is not transparent and the persons evaluated cannot completely reproduce their RG score [7]. Furthermore, it is possible to achieve a high RG score without having a single scholarly publication since moderate social activity is sufficient and it is even possible to add highly cited publications by other authors to one’s own profile [8].

Due to the dynamic publication habits in the different disciplines, little research has yet been undertaken with respect to the relevance of platforms such as ResearchGate etc. to the work of academics [2,9,10]. A consideration of the classical bibliometric indicators related to journal publications is often very one-sided and only reveals part of the impact of scientific publications on society going beyond the classical peer review process [11]. Altmetrics are gaining significance in this context since they are intended to close this gap. The term altmetrics was first coined in 2010 [12] and there is still no generally recognized definition for this approach to measuring academic impact [10]. In addition, there is no consensus in the scientometric community on the value of altmetrics and what information such metrics could offer. In order to approach this question, a survey was implemented among 695 researchers from various disciplines. The survey was conducted prior to the recent actions of the publishers’ “Coalition for responsible sharing” against ResearchGate which are therefore not a subject matter of this publication (More on https://www.nature.com/news/publishers-threaten-to-remove-millions-of-papers-from-researchgate-1.22793 and http://www.responsiblesharing.org/coalition-statement/). Attention was rather focused on usage patterns and the subjective perception of ResearchGate as a social networking site which is currently regarded as the most frequently used platform of its type [3]. Do men or women tend to use ResearchGate more and what role is played by the academic discipline? Can any country-specific differences be identified with respect to ResearchGate’s metrics and what is the subjective perception of the survey participants concerning these metrics? These and many other issues were investigated in a survey consisting of 19 questions. This paper focusses on the following outlined survey concerning ResearchGate. A general inquiry of literature regarding this topic can be found in a separate Altmetrics feasibility study which was also published by the authors of this study [13].

## Research questions/methodology

### Research questions

With the aid of the survey performed for this paper, it should be possible to answer the following questions:

1. How much time do scientists spend using ResearchGate each week and how do they estimate the effort required?
2. What usage patterns can be identified? Which areas of ResearchGate are used more and which less frequently?
3. What do scientists regard as the added value obtained by using ResearchGate?
4. Does the social influence of superiors or colleagues play a part in using ResearchGate?
5. What behavioural intention do scientists have for the future with respect to ResearchGate?
6. What is the significance of the RG score for scientists and what is their opinion of this score?

### Survey model

**Fig 1:**
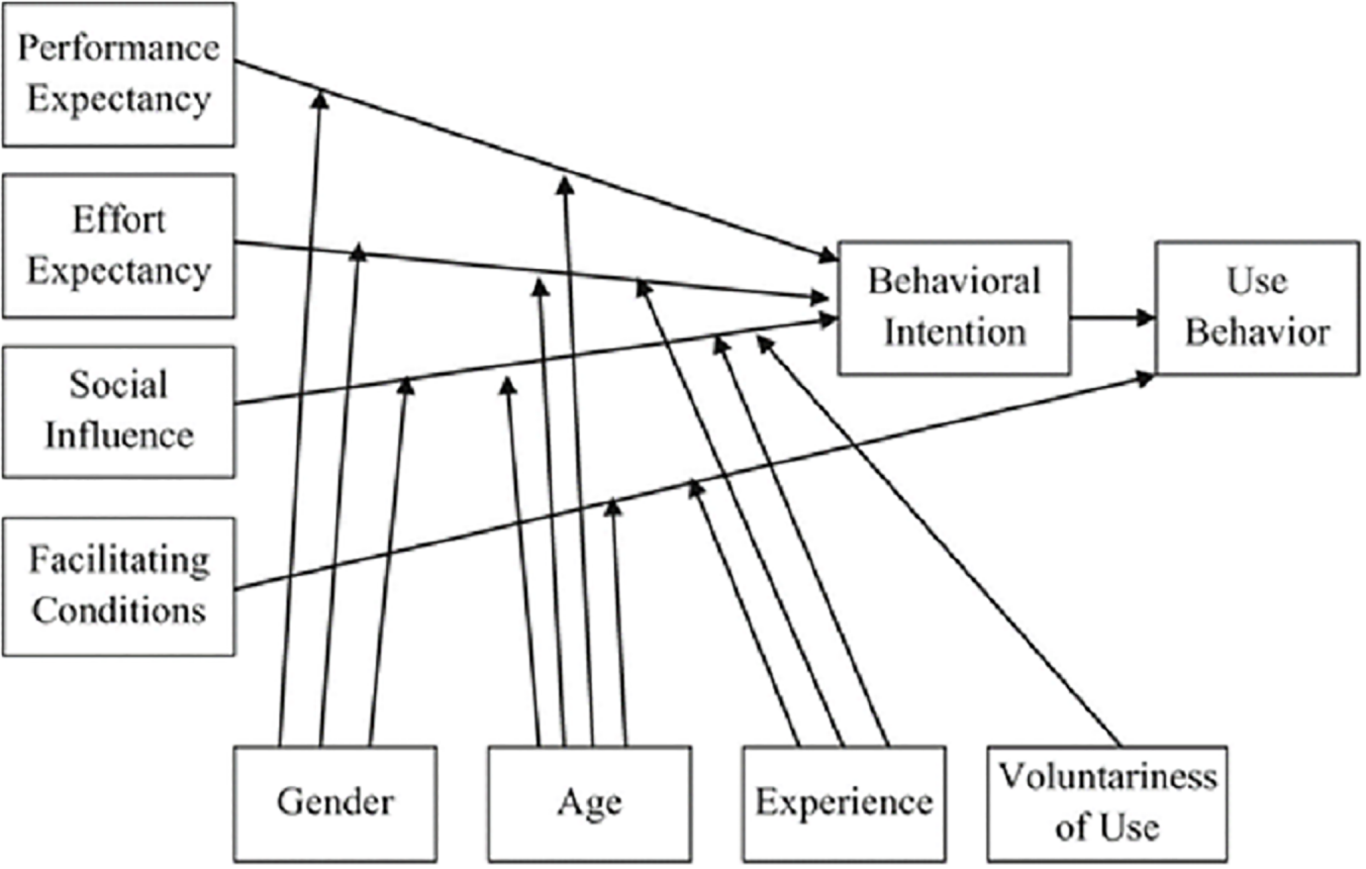
The unified theory of acceptance and use of technology model [13].

The survey model is based on the unified theory of acceptance and use of technology (UTAUT), which is employed to construct user surveys on new information systems. These surveys are intended to help administrators, managers, and executives in companies, universities, or other organizations to estimate how probable it is that a newly introduced system at their institution will be accepted and used [14]. The factors incorporated in the survey were graphically modelled by the authors and are shown in Fig 1. Using this model, as applied to a user survey, will help to discover which behavioural intention and which actual use behaviour on the part of the users of a system can be identified and analysed. As can be seen in Fig 1, several factors are of significance and these factors also influence each other. These factors can be divided into two distinct groups, on the one hand the user’s personal background (*Gender*, *Age*, *Experience*, and *Voluntariness of Use*) and, on the other hand, administrative, social, and personal circumstances (*Performance Expectancy*, *Effort Expectancy*, *Social Influence*, and *Facilitating Conditions*), which may also influence use of the system.

*Performance Expectancy* is intended to measure users’ expectations with respect to the improvement in professional performance which, in their opinion, would be provided by the system to be investigated. Another decisive factor is *Effort Expectancy*, that is to say how easy the system is to use and the effort required. Both factors will be additionally influenced by the gender and age of the interviewees (*Effort Expectancy* is also influenced by the experience the user has already gained with the system). Furthermore, *Social Influence* is intended to reveal whether the user questioned in the survey has been influenced in their use of the system by other persons (colleagues, superiors). As an additional factor, *Voluntariness of Use* plays a decisive role in order to identify the extent to which the respondent uses the system voluntarily. In addition, the remaining personal background (*Gender*, *Age*, and *Experience with the System*) will also have an effect on the social influence. The fourth and final factor, which only influences actual use and is in turn influenced by the age and experience of the user, is *Facilitating Conditions*. This comprises the technical and organizational infrastructure provided both by the system as well as the institution for which the respondent works. The decisive aspect is the extent to which users are of the opinion that this may be of assistance to them.

Since many of the factors cannot be defined by simple yes/no questions and answers, the respondents are able to define their perceptions as far as possible along a scale. This method is based on the Likert scale, which gives respondents the opportunity to indicate their answer on a scale ranging between complete agreement and complete disagreement [15].

**Fig 2:**
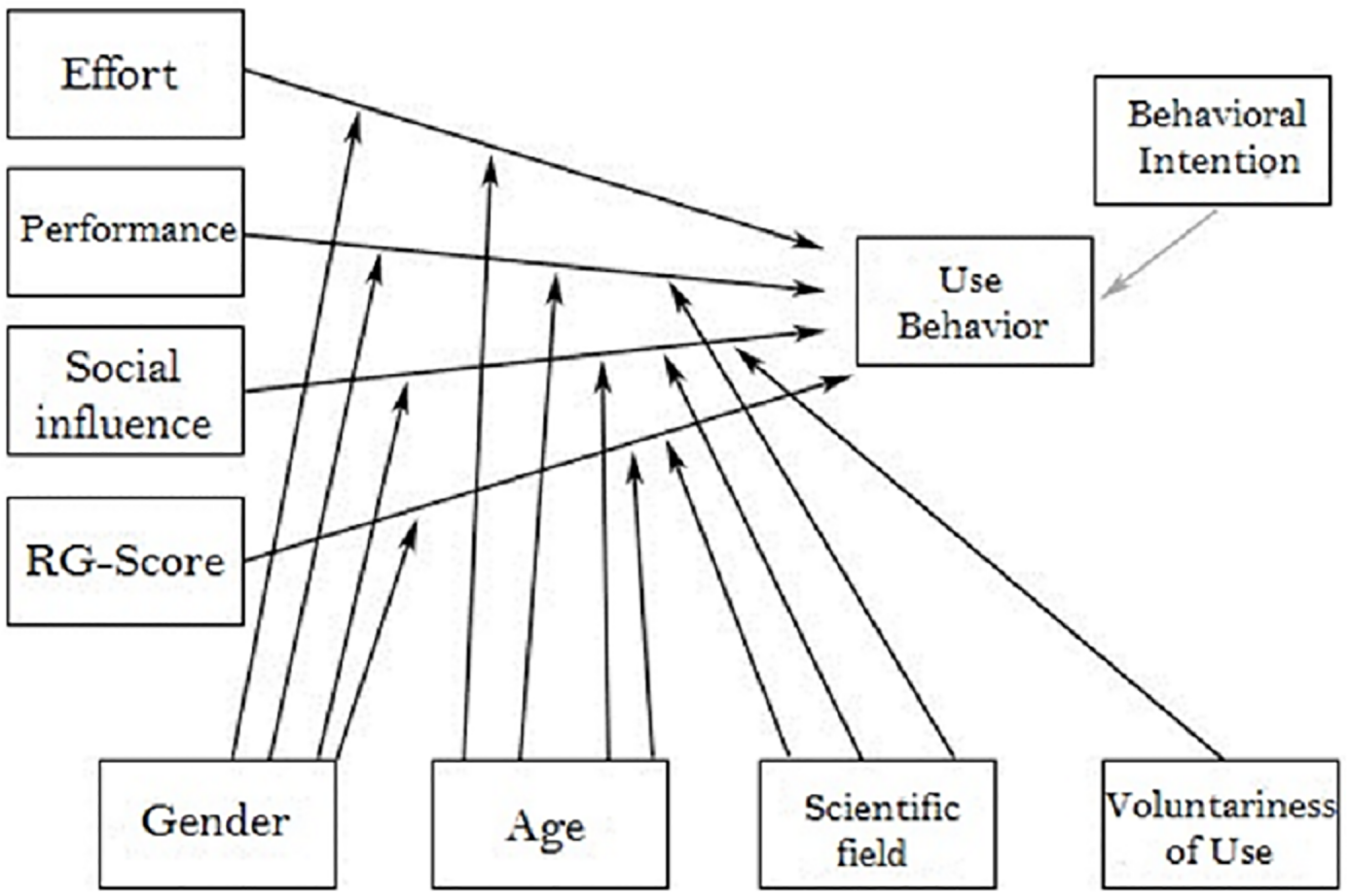
Survey model according to the UTAUT approach of [13].

Having found that the UTAUT model is suitable for the ResearchGate survey, we constructed our own survey model and structure on this basis. To this end, the factors of the UTAUT model were used as far as possible, whereby additional components were added and extraneous factors deleted. The model adapted from the UTAUT approach is constructed as follows (Fig 2).

In constructing the survey model, attention had to be paid that whereas the UTAUT model was designed for newly introduced systems, ResearchGate is a platform that had already been in existence for a number of years. Furthermore, the respondents had been using the platform for various lengths of time so that this factor differed significantly from newly introduced systems for which the period of use would be the same for all participants. This fact has a decisive influence on *Behavioural Intention*, which in the case of newly introduced systems has a primary influence on *Use Behaviour* (see Fig 1). The respondents had the opportunity to estimate their behavioural intention for the new system on the basis of the time during which they had used it. As mentioned above, in the case of ResearchGate due to the different usage periods this is not the case so that the behavioural intention has rather a peripheral influence on actual use (see Fig 2). For the same reason, the factor of *Experience* was deleted because although the question could have been posed, it was expected that the respondents’ answers would have been too imprecise since their period of their membership is not recorded in their profiles. Instead of this, in the components of personal background a question was asked concerning the *Scientific Field* of the participants (Fig 2) in order to determine whether this had an influence on the administrative, social, and personal circumstances. In the final group of factors, the *Facilitating Conditions* were dispensed with since as a system ResearchGate is not an integral part of a scholar’s work and it is generally used on a voluntary basis. The extent to which the voluntariness of use is restricted is taken into consideration by a question concerning the *Social Pressure*. Instead, the *RG Score*, an impact factor indicator specifically introduced by ResearchGate, is included as a significant factor. Amongst other aspects, this concerns the RG score of the respondents as well as their opinion of it.

In the following, the complete survey is presented together with the individual factors and possible responses. The possible responses are given in brackets after each factor or question. If the participants were asked to indicate their estimates on a Likert scale (five points from “totally disagree” to “totally agree”), these questions are labelled *LS*. If the respondents could enter a free answer in a text field these responses are labelled *FA*. Furthermore, respondents always had the opportunity to freely comment on all the questions or statements. The following individual components make up the survey as a whole:

### Used questionnaire

1. **Some information regarding yourself**
  *Your age* (30 or less, 31–40, 41–50, 51–60, 61–70, over 70)
  *Gender* (male, female)
  *Which country are you from*? (FA)
  *Scientific field* (FA)
  *Who created your profile/account on ResearchGate*? (I created my profile/account myself; ResearchGate created a profile and invited me to take over it; Other)
2. **Effort**
  1 *How much time do you spend on ResearchGate weekly (Research*, *Browsing etc*.*)*?
  2 *How much time do you spend on the maintenance of your profile/account (Adding your publications*, *general maintenance)*?
    (For both questions: less than one hour; one to two hours; three to four hours, five hours or more)
  3 *In my opinion*, *the required effort for an active use of ResearchGate is too high*. (LS)
  4 *I am being assisted with the maintenance of my ResearchGate profile/account because I cannot deal with the effort on my own*. (LS)
3. **Performance**
  5 *As a result of using ResearchGate*, *I already was able to experience job-related benefits*. (LS)
  6 *I am sure that I was able to increase my academic influence and reputation with the help of ResearchGate*. (LS)
  7 *In my opinion it makes sense to use ResearchGate as a researcher*. (LS)
  8 *Have you ever used the Q&A-area of ResearchGate in order to ask questions*? (I already have asked questions several times; I rarely have asked questions; I have not asked any questions yet)
  9 *Have you ever used the Q&A-area of ResearchGate in order to answer questions*? (I already have answered questions several times; I rarely have answered questions; I have not answered any questions yet)
4. **Social influence**
  10 *I feel being pressured by my colleagues who advise me to use ResearchGate*. (LS)
  11 *I feel being pressured by my superiors/supervisors who advise me to use ResearchGate*. (LS)
  12 *I use ResearchGate of my own free will and not because it was advised by someone else*. (LS)
  13 *I am rather the person who tries to convince my colleagues to join or to be more active on ResearchGate*. (LS)
5. **Behavioral intention**
  14 *I am committed to be more active on ResearchGate in the future*. (LS)
  15 *I wish I could be more active on ResearchGate but I do not have the time for that*. (LS)
6. **RG Score**
  16 *What is your current RG Score*? (FA)
  17 *In my opinion*, *it is obvious how the RG score is calculated and what it represents*. (LS)
  18 *In my opinion*, *the RG score is an appropriate representation of a scientist’s reputation*. (LS)
  19 *To me personally*, *it is important to steadily increase my RG score and to have a high RG score in general*. (LS)

### Conduct of the survey

The platform Google Forms (Information on Google Forms: https://www.google.com/forms/about/) was selected to implement the survey. In order to reach the greatest possible number of scientists, invitations to take part in the survey were sent to email addresses specified by scientists as contact addresses in their publications, large numbers of which were downloaded with the aid of Web of Science. Four scientific areas were selected for the investigation (medicine, physics, biology, and neuroscience). The query output was downloaded, converted separately into Excel tables, all email addresses were copied from the EM (email) column into a separate sheet and all (assumed) disposable email addresses filtered out (In this case, all email addresses were deleted that merely had a numerical sequence before the @). In order to randomize the email addresses, a random number was generated for each email address from column A into column B (The RAND (random number) function was used to create a purely numerical multidigit decimal figure) and then sorted to column B. Finally, the first 2500 email addresses from each of the four scientific fields were selected and an invitation to participate in the survey was sent to each of them.

The results of the survey are presented in two ways in the following. First of all, the general results of all respondents are presented. Since all the participants had the opportunity to comment on almost all the questions, some of the relevant comments are included in the presentation of results. In such cases, reference is always made to a numbered commentary (for example, in the form (I-695) indicating the last of the 695 survey participants). All the respondents’ answers can be found numbered consecutively under this link (10.6084/m9.figshare.7123172). The results are not presented in detail here (differentiated according to age, gender, origin, and discipline) for reasons of clarity. However, in order to investigate the influence of these factors, the statistical significance was scrutinized with respect to the 19 questions. This was implemented with the aid of the ANOVA test of the statistical analysis software SPSS and on the basis of the calculated p-values shows whether the corresponding factor influences the answers given by the respondents or not. A p-value of 0.05 or less represents a high significance and implies that the answers did not arise randomly but are rather decisively influenced by the related factor [16]. For example, if the factor “Origin” has a p-value of < 0.06 with respect to question 16 (What is your current RG score?), then the RG score is–in a statistical sense–very dependent on the origin of the survey participants. The higher the p-value, which can reach 0.999, the lower the influence of the factor. For our example, a high p-value would mean that there were no parallels at all between the participants’ origin and their RG score.

## Results

In the period from 2 February to 11 April 2016, a total of 695 fully completed surveys were received, after which the survey was closed.

### Information regarding the participants

67% of the 695 participants were men and 33% women. The survey was completed with a relative majority of 35% by 31- to 40-year-olds whereas the proportion of persons over 70 only amounted to 2% and thus represented the smallest age group. Most participants came from Germany (71), followed by the United Kingdom (63), the USA (58), Italy (55), France, and Spain (42 each). In response to the supplementary question of who had created their profile on ResearchGate, 80% stated that they had created the profile themselves, whereas ResearchGate had created the account for 16% of the participants (4% specified other reasons, mostly that the origin of the profile was unknown). It was thus revealed that every 6th profile was not created by the users themselves but by ResearchGate and then subsequently taken over by the scientists.

### Effort

**Fig 3:**
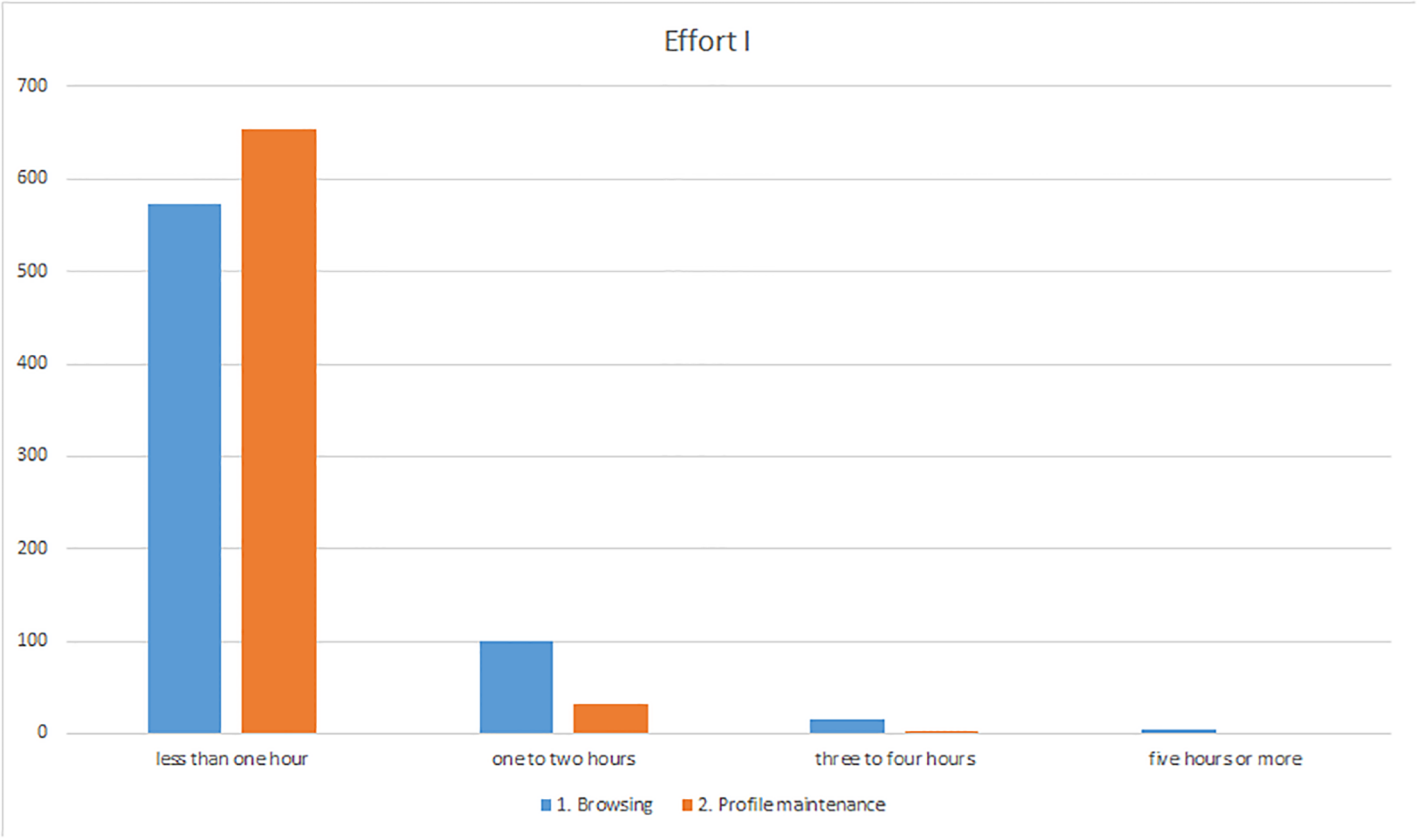
Responses to the “Effort” section (questions 1–2).

**Fig 4:**
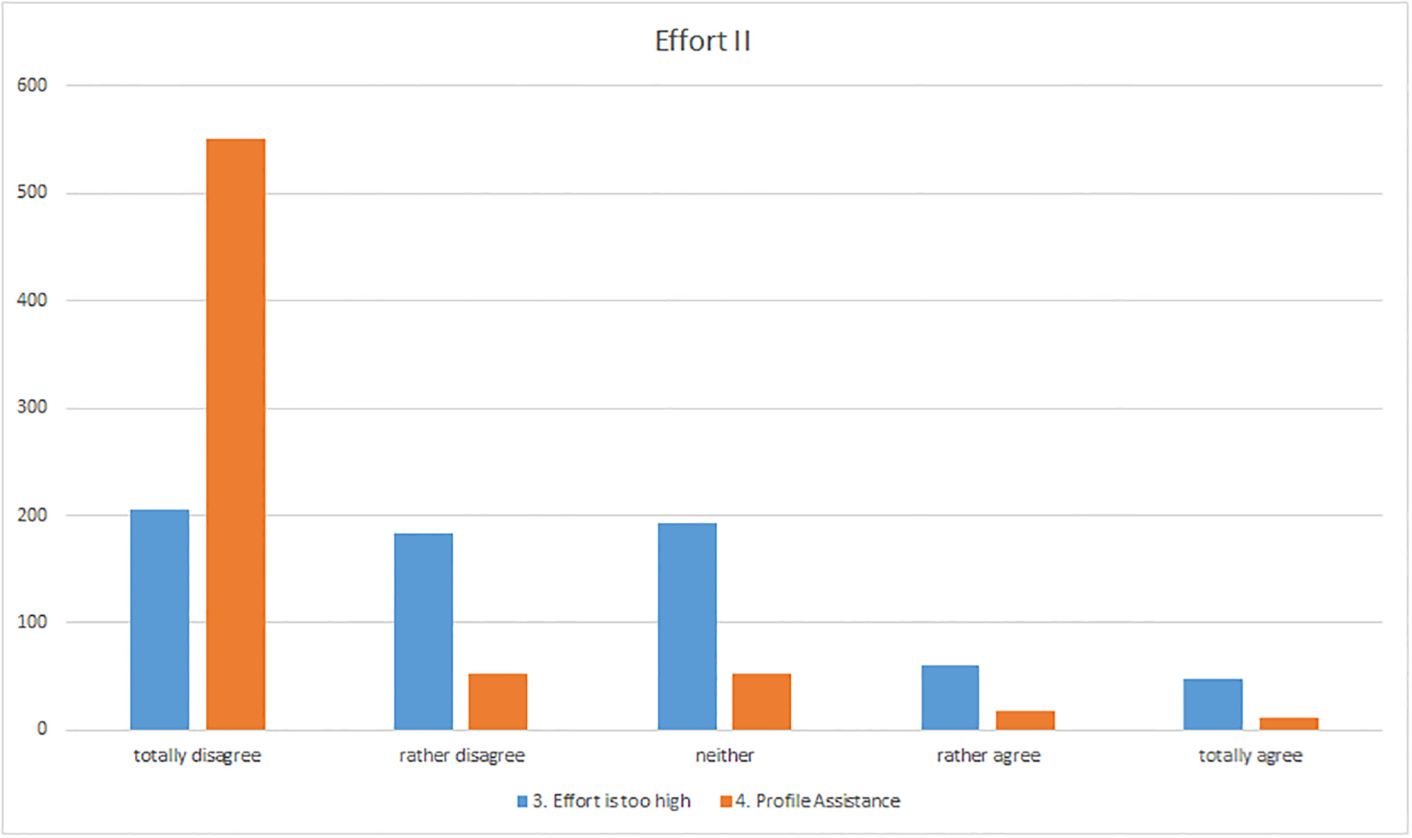
Responses to the “Effort” section (questions 3–4).

The effort invested in ResearchGate by the survey participants seemed to be very low (Fig 3). The majority of respondents said that they spent less than one hour a week on ResearchGate for searches, browsing, or similar activities (83%). Some of the participants stated that the low amount of time they expended was due to the fact that they rarely conducted searches with ResearchGate (I-14) and preferred to use other sources (I-127). Merely 15% of the respondents said that they were active on ResearchGate for one to two hours a week, while only 3% stated they used the site for three to five hours per week or even longer. Even less time was invested in updating their profiles. 94% of the users stated that they spent less than an hour per week on this activity. The reasons included the fact that ResearchGate itself undertook some updating of the profile since publications frequently did not have to be entered by the users but were rather selected by ResearchGate and only had to be confirmed by the users so that these publications could be added to their profile (I-189, I-517, I-679). Most of the survey participants said that they tend to “totally disagree” or “rather disagree” with the statement “In my opinion, the required effort for an active use of ResearchGate is too high.” 55% of users were of the opinion that they did not feel the effort was too great whereas only 16% agreed partly or totally with the statement (Fig 4). This reflected the results from the two preceding questions where it became apparent that the effort expended was very low and only a minority of users felt that demands on them were too great. Furthermore, most respondents did not consider that the system made excessive demands since “some of the work is done for you, such as entering publications” (I-528) and that the system itself is very user friendly and easy to use (I-217, I-567). In this context, the final statement in this part of the survey concerning whether participants need assistance in using ResearchGate leads to a clear result namely that 79% of respondents disagree totally (Fig 4). For most of the respondents no assistance is necessary (I-256), some would prefer assistance (I-273, I-693), for example in processing enquiries (I-96).

### Performance

In the section on “Performance”, participants were requested to estimate the extent to which ResearchGate has benefited them professionally to date (question 5), whether they are of the opinion that the site increased their academic influence and reputation (question 6), and whether they believed that using ResearchGate made sense for them as researchers (question 7). Furthermore, they were also requested to state how often they asked or answered questions using ResearchGate (questions 8 and 9).

**Fig 5:**
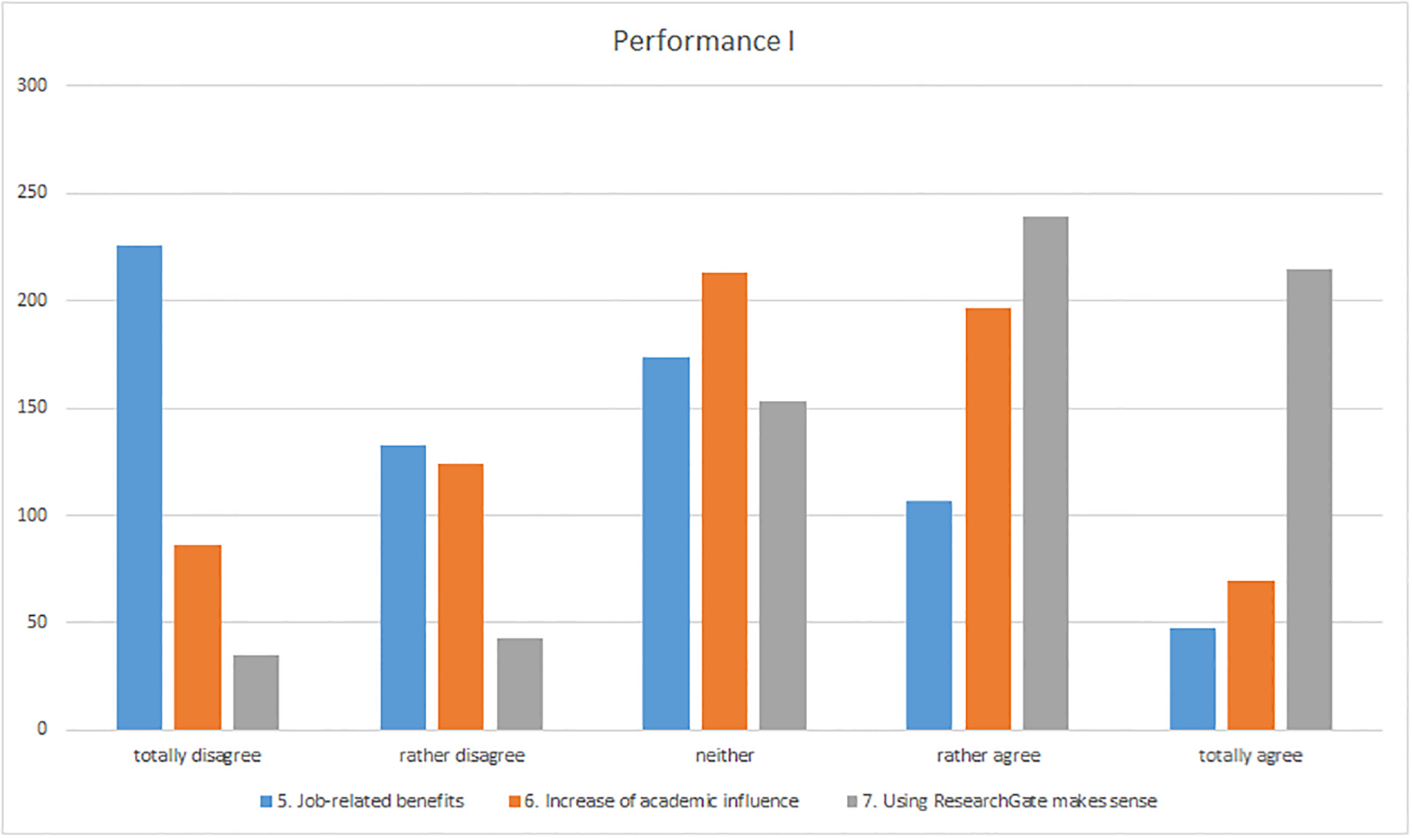
Responses to the “Performance” section (questions 5–7).

Users tended to be in agreement that as yet they perceived no or only few professional benefits from using ResearchGate (Fig 5). The relative majority (33%) totally disagreed with the statement whereas another 19% tended not to agree, and 25% were not certain as indicated by answering “neither”. In her comment, one respondent from the USA tended not to agree with the statement since “It’s nice to see that people are reading my papers, and it’s nice to get alerts when my colleagues publish, but these are no huge benefits in my opinion” (I-164). Many of the participants also stated that they had not been using ResearchGate long enough to judge whether it would be of benefit professionally (I-35) and that “the effects are not immediate” (I-293, I-497). Nevertheless, more than 15% of the respondents tended to agree with the statement that using ResearchGate could provide job-related benefits (only 7% were completely convinced). Free access to scientific articles, which is often not available on other platforms, was frequently mentioned as one of the benefits (I-113, I-223, I-250, I-267, I-426, I-543, I-565, I-575), but also the opportunity to exchange ideas with other scientists (I-207, I-430), and to “have discussions with people I may never meet due to distance” (I-113). One survey participant from India even stated that he had received an offer for a postdoc position through his ResearchGate profile and therefore had already reaped job-related benefits (I-211).

The graph representing the distribution of responses to the statement “I am sure that I was able to increase my academic influence and reputation with the help of ResearchGate” (question 6) indicates a different picture. In contrast to the statement discussed above with respect to job-related benefits, respondents here clearly tended to agree with this statement. While 28% of the survey participants tended to agree with the statement and another 10% were in complete agreement, the proportion of participants who tended not to agree (18%) or disagreed totally (12%) was somewhat smaller. Many of the respondents were convinced that considerably more people would have access to their articles with the aid of ResearchGate (I-200, I-584) and that this had led to a number of collaborations for research work (I-443), which increased their academic reputation. Nevertheless, a large proportion of participants noted that they were uncertain whether they had become better known through ResearchGate and chose “neither” (31%). Some of the survey participants were not certain how this increase could be measured at all (I-16, I-293, I-667). This was in part due to the fact already noted above that ResearchGate is still relatively new (I-35). In contrast, some of the scientists who were not of the opinion that they had already obtained any benefits with the aid of ResearchGate were convinced that they are sufficiently visible on other platforms (I-87) and that established citation databases such as Web of Science or Scopus “were more important in increasing academic profile and influence” (I-152). Furthermore, one respondent from the UK noted that “I have never heard anyone refer to ResearchGate in any professional context, so I think its influence is minimal” (I-160). In this context, some survey participants also noted that they had no interest at all in improving their academic influence and reputation with the aid of ResearchGate (I-248, I-368, I-563).

There was even greater agreement with the statement that it made sense for scientists to use ResearchGate. A total of 34% of the participants tended to agree and 31% agreed totally (Fig 5). Agreement with this statement was also frequently justified by the fact that ResearchGate makes it easier to access scientific material (I-200, I-218, I-679), but also because it was possible for colleagues to answer questions (I-379, I-468, I-639). In comparison, only 11% of the respondents tended to disagree or disagreed completely. Some of them criticized the fact that ResearchGate is merely “another thing to make grad students and postdocs waste time” (I-160) or that in general “time spent with social media means that time is not spent on useful work” (I-552). Furthermore, one participant reduced ResearchGate to “just” a social networking site and criticized that “no real outcome or real community [is] based on it” (I-589).

**Fig 6:**
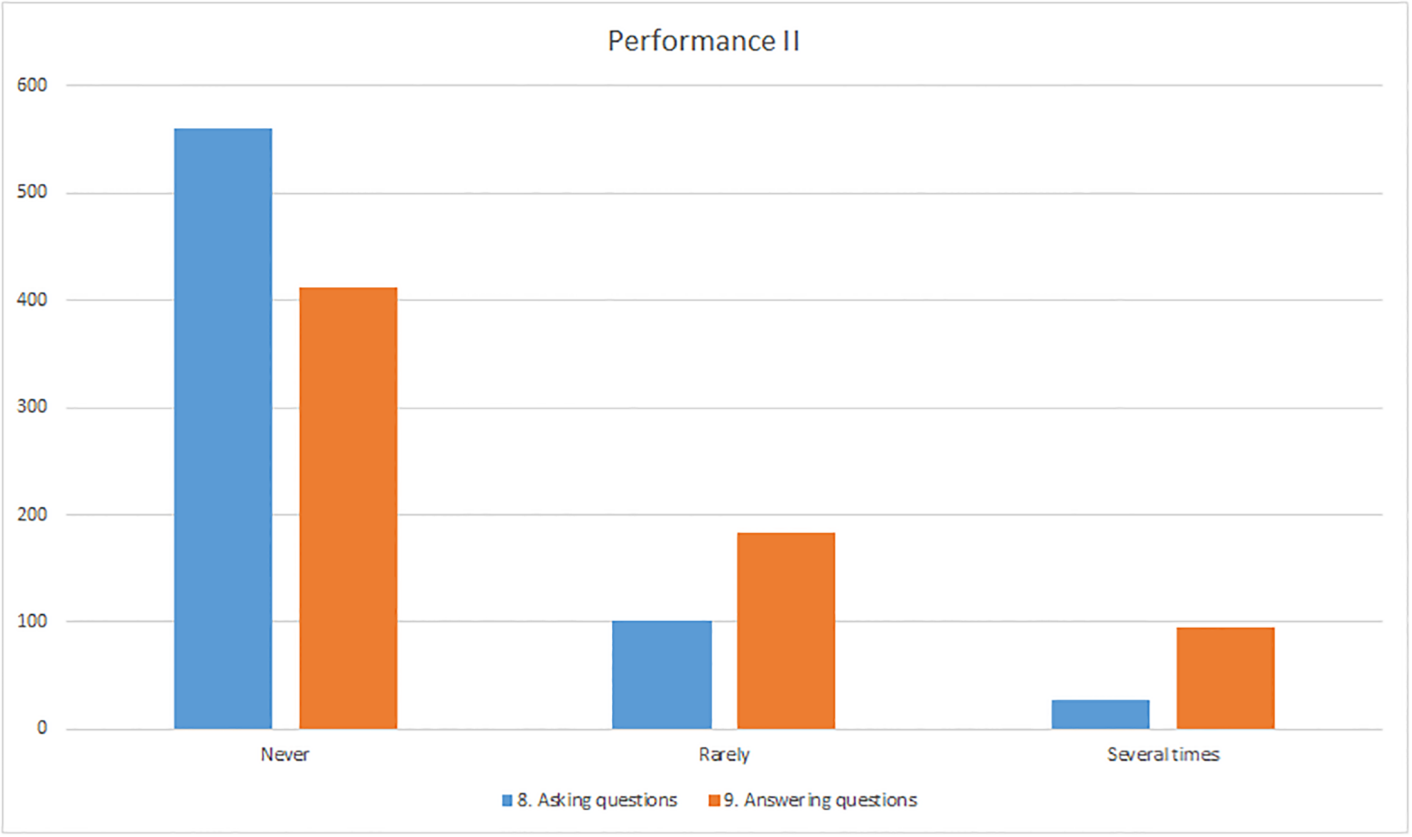
Responses to the “Performance” section (questions 8–9).

The question of how often participants have asked and answered questions on ResearchGate received a variety of answers. 81% of the respondents had not yet asked any questions and 59% had not answered any. The respondents were less active in asking questions than in answering them: 19% said that they had asked questions rarely or several times whereas 41% had already answered questions (Fig 6).

**Fig 7:**
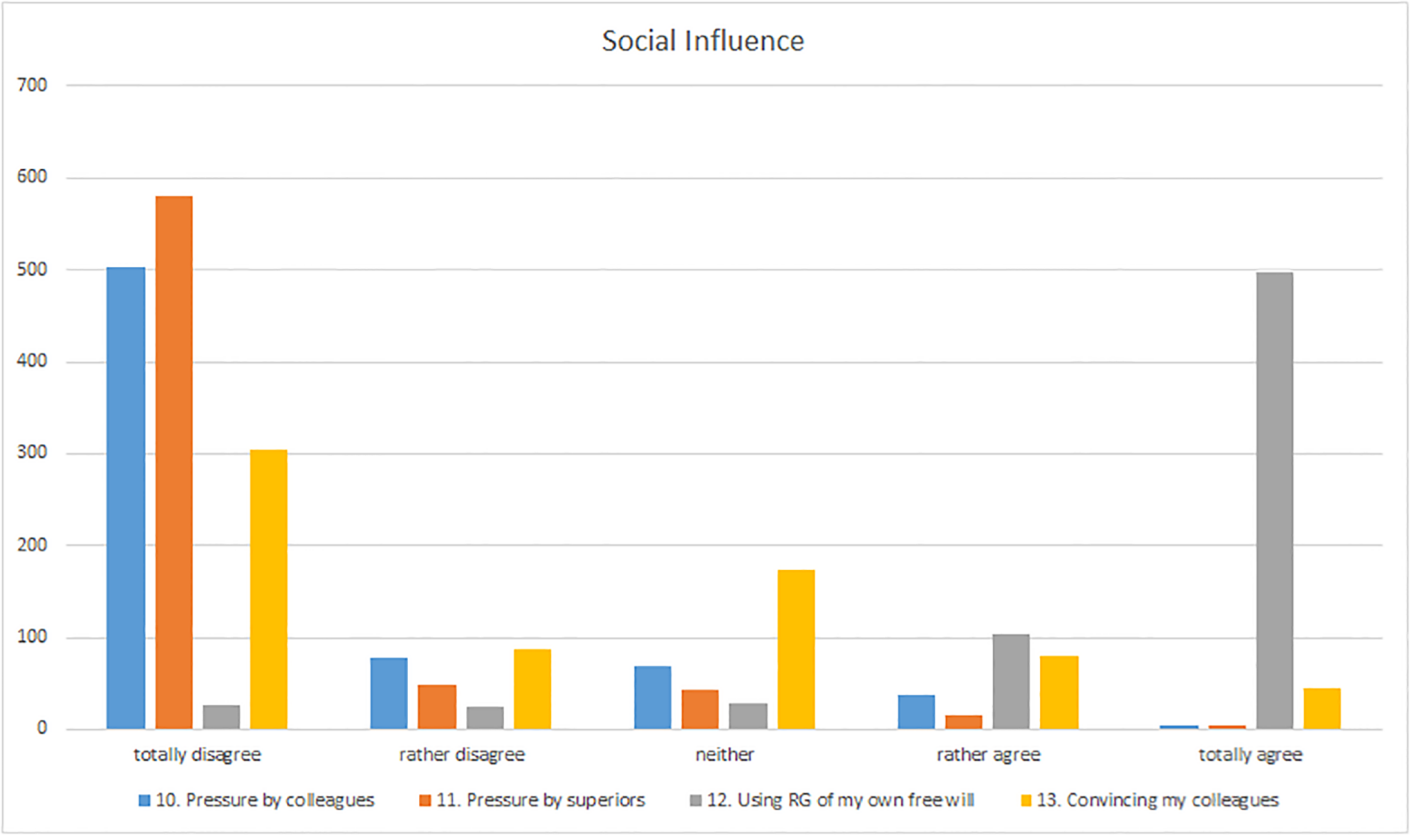
Responses to the “Social influence” section (questions 10–13).

The vast majority of participants did not feel they were being pressurized by colleagues or superiors to use ResearchGate (“Social influence” section, questions 10–11). Only 6% of the respondents tended to agree or agreed totally that they felt they were being socially pressurized by their colleagues (Fig 7). This was manifested by, for example, emails urging them to register. Participants resorted to labelling such invitations as spam (I-596). However, the majority of participants tended to disagree or disagreed totally with this statement. Some of the respondents commented that in their environment ResearchGate had either never been mentioned (I-113, I-160, I-688) or that nobody would take it seriously (I-391, I-552).

### Social influence

The response concerning the subjective perception of social pressure by superiors is similar. Nine out of ten respondents tended to disagree with this statement or disagreed completely. The reasons included the opinion that the respective superiors were either not aware of ResearchGate (I-273) or they ignore it (I-552) so that no social pressure was exerted from above. Furthermore, it was occasionally noted that ResearchGate had been mentioned (I-113) or recommended (I-241) by superiors, but without any pressure. As far as using ResearchGate is concerned, only 3% of the international survey participants were of the opinion that they felt under any sort of social pressure (Fig 7). No comments were found that could provide any examples or any reasons for this.

This perception was also reflected in the third statement concerning social pressure with respect to whether ResearchGate was used voluntarily by the participants and not because somebody else wanted them to. More than 77% of the respondents said that they tended to agree or totally agree with this statement (Fig 7). In this case as well, one survey participant stated that he did not experience any pressure to use ResearchGate and that use was entirely voluntary (I-391), whereas others are of the opinion that although use is voluntary “Research Gate itself is a bit aggressive with their constant emails to get one to sign up” (I-677). However, precisely for this reason one participant did not agree with the statement since “I would have never used it without being bothered by requests” (I-368). Altogether, only 7% tended to disagree or disagreed totally with the statement that use of social networks is on a voluntary basis.

With respect to the question of whether it was rather the person him- or herself who tried to persuade their colleagues to use ResearchGate more actively, most respondents tended to disagree or disagreed totally (Fig 7). Whereas in both cases, the relative majority (56%) tended not to agree with the statement or disagreed totally, nevertheless 18% of the respondents tended to agree with the statement or agreed totally. The main reasons why the survey participants did not appear to recommend their colleagues to use ResearchGate included the fact that ResearchGate was not a topic of conversation between colleagues (I-308, I-550) or that social networks were regarded as unimportant or even a waste of time (I-451, I-550, I-563). Those persons who tended to persuade their colleagues that they should actively participate in social networks did so because they were convinced that, for example, “researchers who do not have active and accessible digital profiles (not only on RG) are in danger of being less visible to the community” (I-109) or because “it’s a good platform to exchange the latest papers from people in your field” (I-528). One respondent from Libya even said that he advised every scientist to register with ResearchGate (I-662).

### Behavioural intention

**Fig 8:**
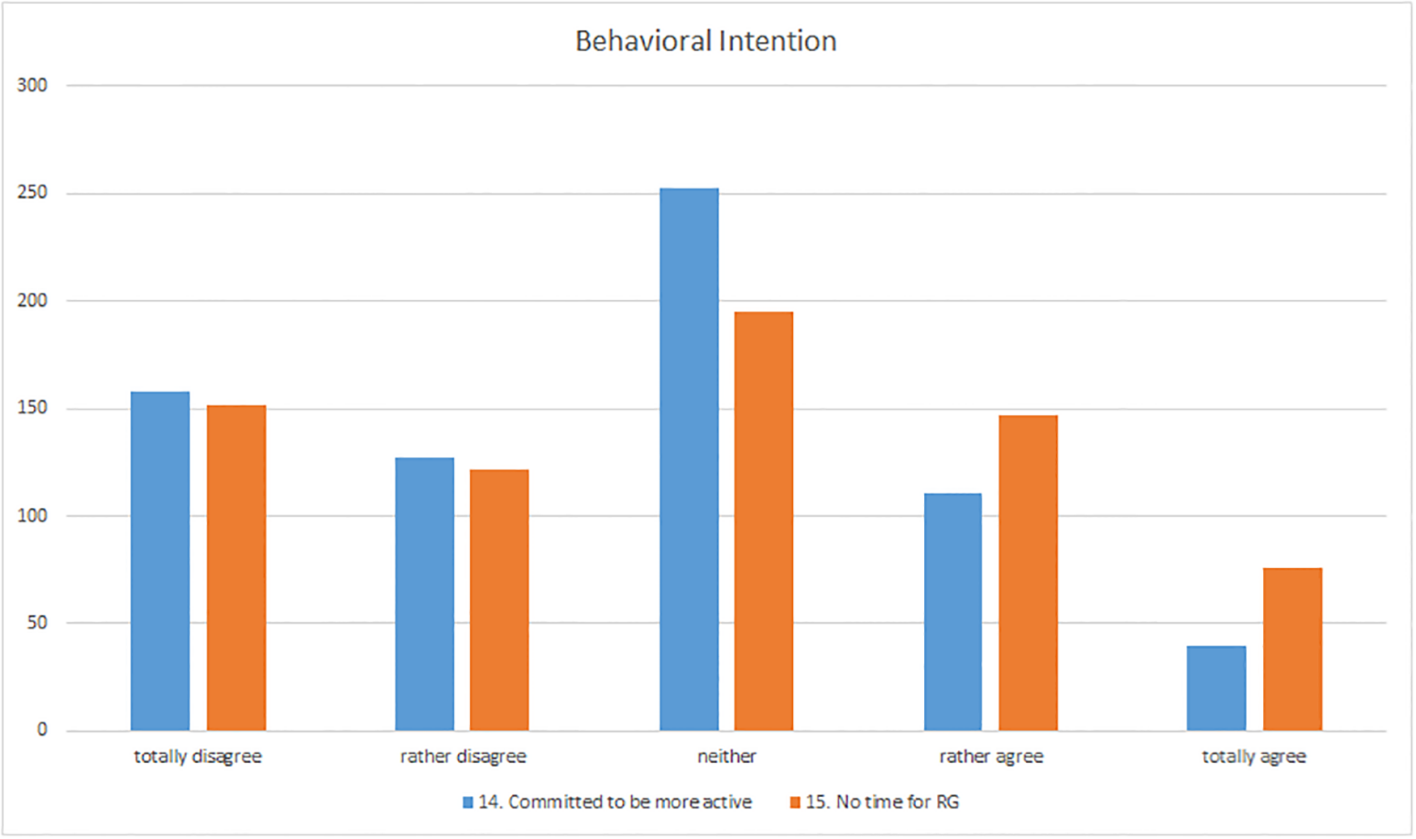
Responses to the “Behavioural intention” section (questions 14–15).

Concerning the respondents’ behavioural intentions with respect to using ResearchGate, the majority said that they intended to use ResearchGate no more and no less in future: 42% of them responded to statement 14 “I am committed to be more active on ResearchGate in the future” with “neither” (Fig 8). Some of them commented that they intended to remain as active as they currently were (I-189, I-430) or that increased activity on ResearchGate would not further benefit their career (I-528). More than 22% agreed totally with the statement. Nevertheless, rejection of more intensive use of ResearchGate was the majority view although 42% of the respondents tended not to agree or disagreed totally. One reason specified was that they saw no point in using it (I-160), that they didn’t see any benefit in being more active (I-421), or “Maybe, if the website improves” (I-131).

Statement 15 concerning whether users would like to be more active on ResearchGate but did not have the time received somewhat more approval (Fig 8). 23% tended to agree or agreed totally with the statement. One respondent commented that lack of time was the reason for his limited use (I-549), whereas another respondent was of the opinion that “There are so many things that take our time up as researchers. Everyone I know has ‘information overload’” (I-517). However, although there was more agreement with this statement than with the previous one, the majority of respondents still rejected the statement. The relative majority of 28% of the respondents was not certain whereas another 40% of them tended not to agree or disagreed totally. One participant from Norway stated “I do not have time—this part is true. But I would not be very interested in being more active on it even if I had more time, I think” (I-13). Other respondents also stated that they would not be interested in using ResearchGate more actively in future (I-160, I-217, I-563).

### RG-Score

**Fig 9:**
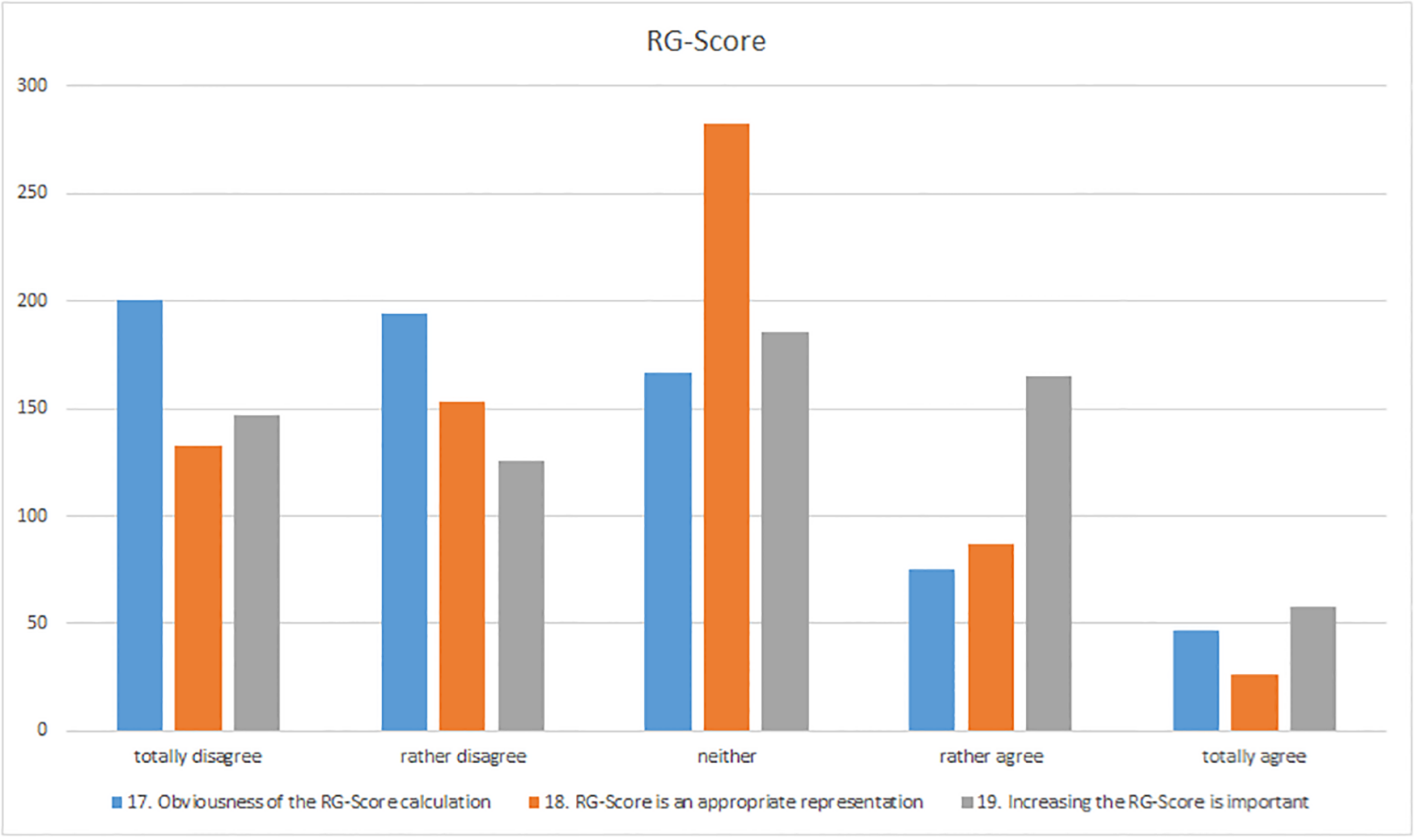
Responses to the “RG score” section (questions 17–19).

How the RG score is actually calculated and what exactly this metric represents does not seem to be clear internationally (Fig 9). More than half the survey participants (57%) stated that they tended not to understand or didn’t understand at all how the ResearchGate metric was calculated. Many were of the opinion that the calculation is not only unclear (I-413, I-453, I-507, I-549, I-616, I-661), but also that it was not properly explained by ResearchGate (I-93, I-236), and that “It’s totally in-transparent, nobody has the slightest idea how this score is calculated” (I-224). Many other participants tended not to agree or totally disagreed because they had no interest in the RG score or because they were completely indifferent to it (I-13, I-14, I-160, I-169, I-200, I-233, I-387, I-412, I-439, I-679). Two of the participants actually stated that they were unaware of the RG score and had only heard about it in the present survey (I-374, I-567). Furthermore, many of the participants were of the opinion that other metrics such as the h-index (I-13, I-207, I-224, I-391) were clearly superior to the RG score because they are more transparent and easier to calculate (I-13, I-224) and in any case “the H-index is more important and more widely used” (I-391). In contrast, only 18% were convinced that they understood the significance of the RG score and how to calculate it. However, whereas many of the participants who rejected the RG score provided comments on their answers, no comment was included by those who approved of the score which might have indicated why they were of this opinion.

The survey participants seemed to be uncertain about the role of the RG score and whether it adequately represents the scientific reputation of a researcher (Fig 9). In the survey, the relative majority (41%) of participants selected the response “neither”. Some of the respondents who were unable to agree or disagree were of the opinion that they were not quite clear about what the RG score was intended to represent (I-16, I-71, I-88, I-154). Some participants were also uncertain due to the problem that the calculation is not transparent (I-164, I-246, I-366, I-439, I-535, I-574, I-584). In this case as well, the opinion was expressed that the h-index would be a better choice for representing the scientific influence of a researcher (I-132, I-680). Due to the fact that answering questions also influences the RG score, one respondent was not certain of the extent to which “the amount of time a scientist has spent answering questions is indicative of the reputation” (I-526). Another criticism was that some users faked data about their publications in order to inflate their RG score and that ResearchGate had no measures in place to identify this (I-418). In general, the survey participants tended not to be of the opinion that the RG score is a suitable metric for representing the reputation of a scientist in one figure. Only 17% of the respondents tended to agree or agreed totally whereas 41% tended not to agree or disagreed totally. However, in this case as well, similar to the question of how the RG score is calculated, none of those who agreed with the statement gave further information on their opinion in the comment box. One respondent who was in agreement merely commented “I guess it is. But I use H-Index as it’s more conventional” (I-131). The rather inadequate justification was also put forward concerning the RG score that it “must be relatively realistic, because people seem to care” (I-368). However, reasons for a rejection of the metric appear much more frequently in the comment boxes. In the same way as the participants who responded with “neither”, respondents also criticized, but more emphatically, the fact that social activity in the form of questions and answers influences the RG score (I-1, I-120, I-156, I-236, I-241, I-572, I-582, I-639, I-665). However, not only the RG score but also other parameters such as the h-index or the impact factor are viewed critically. The reputation of a researcher cannot simply be expressed in one single figure (I-10, I-93, I-113, I-251, I-256, I-552) or “quantified” in general (I-169). One participant from Mexico asserted “there is not such a thing like ‘appropriate representation of a scientist’s reputation’” (I-379). Another respondent was of the opinion that “the use of algorithmically derived metrics to evaluate the merit of anyone is highly suspect. It is a very bad trend that will only end in tears” (I-554).

In spite of the widespread rejection of the RG score and scepticism about its general value, the respondents’ personal and subjective appreciation of their own RG score seems to be more important (“To me personally, it is important to steadily increase my RG score and to have a high RG score in general”, question 19). Only 21% disagreed totally and another 18% tended to disagree, whereas 27% (i.e. one quarter of the participants) were uncertain. Nevertheless, 24% of the respondents tended to agree and 8% agreed totally that their RG score was important to them (Fig 9)– 32% in total. The reasons for this agreement include the opinion that increasing one’s RG score is a sort of competition for the users (I-650, I-680) and that it is mainly “only for fun” (I-490). Others even go a step further and are of the opinion that the RG score “will motivate me to do my best” (I-662), while another participant believed that the RG score will help him since “I want to be one of the best in my chosen field” (I-576). Furthermore, one participant from the field of psychology valued her RG score “just because it could help my personal self-esteem” (I-207). One respondent explained his agreement by saying "I guess some people care, so higher score probably can’t be worse" (I-200). Another respondent stated rather negatively that “probably looks good, but the score is just an ego thing” (I-582). In addition, one respondent commented that “luckily the Uni administrators have not yet found a way to use the RG score to assess our performance” (I-601). If this were the case, some participants would assign considerably more significance to the RG score ("…if I were up for promotion and my chair/dean valued it, I would work to improve it", I-364; "Not until funding bodies ask for it", I-233).

### ANOVA-Test

In the following, the results of the ANOVA test of the statistical analysis software SPSS are presented (Table 1). This analysis is used to determine which factors have a statistical influence on the respondents’ answers and which do not. Here, the mean values are generally used as a reference in order to represent the answers of the survey participants. Responses are quantified on the Likert scale by 1 (totally disagree) to 5 (totally agree). Thus, the higher the mean value the greater is the agreement of the group of participants with the respective statement. Questions 1 and 2 (effort) as well as 8 and 9 (asking and answering questions) were also quantified for the ANOVA test. Similarly, the higher the mean values, the greater the effort the participants have invested in asking or answering questions.

**Table 1:** Influence of the factors of age, gender, origin, and scientific discipline on the questions in the survey about ResearchGate on the basis of the statistical significance (implemented by the ANOVA test of the SPSS statistical analysis software (a significance of 0.05 or less indicates that the factor has a decisive influence on the participants’ answers)).

	Age	Gender	Origin	Discipline
**1. RG_Browsing**	0.133	0.516	**0.002**	0.406
**2. RG_Profile**	0.533	0.865	0.138	0.317
**3. Effort_TooHigh**	**0.021**	**0.012**	0.094	**0.032**
**4. Assistance**	0.573	0.208	0.094	0.554
**5. Job_Benefits**	**0.014**	0.523	0.172	0.111
**6. Academic_Influence**	0.405	0.918	**0.000**	0.067
**7. Using_RG**	**0.003**	**0.007**	**0.000**	0.406
**8. Asking_Questions**	0.776	0.271	**0.000**	0.135
**9. Answering_Questions**	0.158	**0.006**	0.072	0.465
**10. Social_Influence_Coll**	0.819	0.610	0.164	0.877
**11. Social_Influence_SV**	0.275	0.159	0.400	0.908
**12. Social_Influence_FreeWill**	0.684	0.808	0.256	0.881
**13. Social_Influence_ConvinceColl**	0.228	0.486	**0.001**	**0.025**
**14. Intention_MoreActive**	**0.003**	0.085	**0.000**	**0.016**
**15. Intention_NoTime**	0.355	0.129	**0.002**	**0.015**
**16. RG_Score**	**0.000**	**0.015**	0.949	0.156
**17. RG_Score_Calculation**	0.789	0.203	**0.000**	0.135
**18. RG_Score_Representation**	0.707	0.859	**0.000**	0.584
**19. RG_Score_Importance**	0.938	0.251	**0.000**	**0.035**

Altogether, it can be established that the factor “origin” has the greatest influence on the responses of the survey participants: ten out of 19 questions display a p-value of less than 0.05 and thus very high statistical significance while three other questions are very close with a p-value of < 0.1. The scientific discipline and the age group are represented by five p-values each under 0.05 out of a total of 19. The former factor is also represented by another p-value of < 0.1. Of the four factors, gender seems to have the lowest influence on the responses and only has a p-value of less than 0.05 for four out of 19 questions (however, with another p-value of below 0.1).

In the first block “Effort” (questions 1 to 4), above all question 3 concerning the subjective perception of effort indicates that personal factors seem to exert an influence. Age, gender, and scientific discipline display a very high statistical significance (p-value < 0.05), whereas origin remains just below 0.1. Among the age groups, it can be seen that the older the participants are, the greater the effort they perceive in using ResearchGate. Whereas the participants under 30 years of age display a mean value of 2.28 on the Likert scale, the value for the 61- to 70-year-olds is 2.80 (the mean value of all participants is 2.37). Women (mean value 2.20) seem to find that using ResearchGate involves less effort than men (mean value 2.44). Different tendencies can also be identified among the scientific disciplines. Neuroscientists display the lowest mean at 2.20, whereas medical scientists have the highest mean value at 2.55 and of the disciplines investigated in the survey they thus rate using ResearchGate as involving the greatest effort.

With respect to the first question of how much time they expend on searches and browsing on ResearchGate, a high statistical significance can only be determined for the factor of origin. Participants from Canada seem to spend the least time using ResearchGate (mean value of 1.05 on the Likert scale), followed by the USA (1.07), the Netherlands and the United Kingdom (both 1.10). Respondents from Italy (1.29), India (1.37), and Spain (1.38) also on average invest relatively little time in ResearchGate, but somewhat more than participants from Canada, the USA, and the UK.

The second block concerned with “Performance” is the one most greatly influenced by the factor of origin. For questions 6 to 8 a p-value of 0 is calculated (for question 9 a p-value of 0.072) and they thus display the greatest or at least a high statistical significance. Participants from India (mean value of 3.53), Brazil (3.50), and Spain (3.16) clearly tended to be of the opinion that their academic influence and reputation could be increased by using ResearchGate (question 6) rather than scientists from the USA (2.54), France (2.64), and the United Kingdom (2.70). Question 7 ("In my opinion it makes sense to use ResearchGate as a researcher") yields a similar picture. Of the countries investigated, participants from France (3.15), Canada (3.47), and the USA (3.50) agreed less with this statement, whereas respondents from Italy (4.02), India (4.15), and Brazil (4.26) were more clearly of this opinion. The results concerning questions 8 and 9 show that participants from these countries were also most active in ResearchGate’s Q&A section (how frequently survey participants have asked and answered questions on ResearchGate). India displays the highest mean values followed by Brazil and Spain.

The age group is a decisive factor in the participants’ subjective evaluation of whether they have been able to experience job-related benefits from using ResearchGate or whether it makes sense for them to use the network. As regards the latter question, with increasing age the participants seem to be less and less convinced of the benefits since the mean values decrease with each age group. Whereas the participants under 30 years of age display an average of 4.01, this decreases continually and is merely 3.46 for the 61- to 70-year-olds. Although no constant pattern can be identified for the first-mentioned question, the older participants seem to perceive fewer job-related benefits from ResearchGate (1.97), whereas the 31- to 40-year-olds are much more convinced with an average of 2.62.

A discrepancy can be seen between men and women. Whereas more women are of the opinion that it makes sense for them as scientists to use ResearchGate (3.97 in comparison to 3.73 for men), men nevertheless seem to use the platform more often than women as a social media platform to answer questions (1.60 in comparison to 1.44).

Only the factors of origin and scientific discipline of the participants have an impact on the responses concerning “Social influence” (however, only on question 13). In this case as well, it becomes clear that participants are convinced by ResearchGate to a different extent depending on origin. Similar to the previous case, participants from Spain (2.64), Brazil (2.58), and India (2.54) are among the countries with the highest mean values with respect to whether they try to persuade their colleagues to use ResearchGate. In contrast, the USA (1.68), Canada (1.74), and the Netherlands (1.90) attach the least significance to this aspect.

With respect to the same question, there are different opinions depending on discipline. Medical scientists (2.45) and biologists (2.41) attach more significance to persuading their colleagues to use ResearchGate, the mean values are lower for physicists (2.15) and neuroscientists (2.06).

Many high statistical significance values can be identified for questions from the “Behavioural intention” section with respect to the various factors. Origin and discipline have a decisive influence both on the intention to be more active on ResearchGate as well as participants’ personal opinion that they have too little time to do so. In this case as well, respondents from Spain (2.92), Brazil (3.05), and India (3.24) have the strongest intention to become more active, and at the same time they are also most strongly of the opinion that they do not have enough time to do so (Spain 3.08, India 3.09, and Brazil 3.32). Participants from the USA (2.17), Canada (2.26), and Germany (2.27) have the weakest intention of increasing their activity in future. Respondents from these countries are also less frequently of the opinion that this is related to a time problem (Germany 2.30, USA 2.50, and Canada 2.53).

Biologists most strongly intend to use ResearchGate more frequently in future (2.80) whereas neuroscientists express this intention least frequently (2.40). Medical scientists are of the opinion that they have the least time for this social network (3.16). Physicists and neuroscientists (2.68 each) relate their behavioural intentions least frequently to problems of time.

Furthermore, on the basis of the age groups, it can be determined that with increasing age survey participants have less and less intention of increasing their activity on ResearchGate. Whereas the participants under 30 years of age display the greatest tendency to more activity (2.81), this decreases with each age group, and is merely 2.15 for the 61- to 70-year-olds.

With respect to the level of the RG score, there is a clear link to age (p-value 0.000) and gender (0.015) of the survey participants, indicating a very high statistical significance. On average, young respondents (< 30 years of age) have a considerably lower RG score (~13.1) than the older respondents (~39 for the 61- to 70-year-olds), and male participants (~28) specify on average a higher RG score than the women (~24.6). Origin does not seem to have any relevance for the level of the RG score (p-value 0.949) and the scientific discipline only displays a tendency in this direction (p-value 0.156).

With respect to the transparency of the calculation, representation of scientific reputation, and significance with respect to the RG score (questions 17 to 19), origin was always the factor which displayed a p-value of 0.000 for all these questions and thus the highest statistical significance. In this case as well, participants from Spain, Brazil, and India represented the highest mean values. The USA and Canada in contrast were always among the three countries that agreed with these statements least frequently (with Germany, the Netherlands, the United Kingdom, and France as the respective third country).

It was also possible to identify a statistically different opinion with respect to the personal importance of the RG score among the different disciplines. Medical scientists (2.96) and biologists (2.95) attached more value to the level of their RG score than physicists (2.65) or neuroscientists (2.64).

### Overview

In conclusion, the findings show that some tendencies regarding age, gender, origin and scientific discipline can be seen concerning the use of ResearchGate.

#### Age

Older participants tend to experience that the use of the platform needs more effort than it is the case with younger users. Job-related benefits and usefulness linked with the use of ResearchGate are confirmed more by the younger survey participants while the older responders are more sceptical regarding these benefits. In this context, the younger participators have a bigger intention to increase their activity on ResearchGate while this intention decreases with each of the older age groups. Concerning the RG-Score, older participants have a higher average score than younger users.

#### Gender

Men seem to find that the use of ResearchGate involves more effort than women while the latter gender group seems to be more convinced that using the platform makes sense as a scientist. Male participants stated a slightly higher average RG-Score than women. Besides these minor deviations, no compelling differences could be found regarding the gender.

#### Origin

Regarding the factor origin, the most differences can be witnessed in the responses. Users from Italy, India and Spain seem to spend more time on ResearchGate than users from Canada, the US or the Netherlands. Concerning the opinion that academic influence and reputation could be improved by the platform, international differences can also be witnessed: here, participants from India and Spain are also in the same group which supports this opinion while the US users are more sceptical. Together with Canada and the Netherlands, these are also the survey participants that show the least interest in convincing their colleagues to use ResearchGate while Spain, Brazil and India are among those who advise their surroundings to do that. The same group shares the same intention to increase their activity on ResearchGate in the future. With respect to transparency of the RG-Score calculation, the representation of scientific reputation and significance of the score, these three countries also show the most positive attitude while the participants from the USA and Canada are the most sceptical ones.

#### Scientific disciplines

Neuroscientists tend to apply the least effort in using ResearchGate and to persuade their colleagues to use it while medical scientists show in both cases the biggest commitment among the representatives of the scientific disciplines. The personal importance of the RG-Score seems to be more noticeable with medical scientists and biologists while physicists and neuroscientists tend to be less interested in it.

## Discussion of results

The empirical results of this investigation convey an impression of how the ResearchGate social networking site is perceived by its users from the world of science, what value it has in their everyday work, and what role metrics such as the RG score play for scientists.

The survey clearly reveals that the respondents invest relatively little time in browsing ResearchGate or updating their own profiles. Among others, the reasons mentioned for this were preferential use of other sources or the simple and rapid handling of profile updates. The participants tended not to feel that too great demands were made on them by ResearchGate and very few of them had assistance in updating their profiles. With respect to the question of whether they experienced job-related benefits by using ResearchGate or were able to increase their academic influence and reputation, opinions tended to diverge somewhat since although agreement with the first statement was rare, the second statement received considerably more approval. Free access to publications was the most frequently mentioned benefit as well as the opportunity to exchange ideas with other scientists. There are critical opinions concerning whether using ResearchGate increases one’s own influence and reputation since it was difficult to measure this aspect. Nevertheless, the majority of participants are of the opinion that it makes sense for scientists to use ResearchGate. In the Q&A section of ResearchGate, the survey participants seem to be more concerned with answering questions than asking them although the respective majority has never done either. Respondents state that they have hardly experienced any pressure from colleagues or superiors advising them to use ResearchGate and that they largely do so of their own free will. Comments included the fact that ResearchGate had never been a topic of discussion with colleagues or that it was in any case regarded as a waste of time. With respect to behavioural intentions, the survey revealed that the respondents did not intend to increase their activity on ResearchGate, and in any case many of them did not have sufficient time to do so. How the RG score is calculated tends not to be obvious to the respondents: in part due to the inadequate explanation and ResearchGate’s lack of transparency. The opinion that the score provides an adequate representation of the scientific reputation of a researcher also tends to be negative. The main reasons for rejecting the ResearchGate metric include the lack of transparency of the RG score as well as superior alternatives such as the h-index. The participants displayed more interest in their own RG score although it was of little or no importance to the majority of respondents. However, whereas these results are valid for all survey participants, an analysis using the ANOVA test of the statistical analysis software SPSS indicated that the responses frequently depended on the participants’ country of origin as well as in certain cases on their age, gender, and scientific discipline.

If the RG score is considered from a bibliometric perspective, then caution must be exercised in using simplified metrics. As has become apparent with the example of the impact factor, once a metric has been let loose it is very resilient even if it has proved to be unsuitable or false. This can be amply illustrated by the cumulative impact factor, which is frequently applied incorrectly especially in medicine by being attributed to publications by persons. The cumulative impact factor is a simple addition of the journal impact factors (JIF) of a set of publications. Although this procedure is regarded as scientifically false [17], the medical community is not prepared to dispense with the cumulative impact factor. On the contrary, it is used, for example, in official professorial appointment procedures.

The RG score ultimately has no informative value as a composite indicator since it combines two completely different dimensions: on the one hand, the level of the citation of publications and, on the other hand, the participation of persons in social communication. There is no connection between these two levels. That is to say, without a single publication of one’s own it is possible to achieve a by no means insignificant RG score simply by participating in social communication (see e.g. [8]). It is not so easy to capture what is measured on the level of social communication and how it is designated or interpreted. In any case, it becomes apparent that with the RG score the percentage relation of the composition of the score (these percentages can be viewed on the website) should not be neglected in order to gain an impression of whether the RG score is mainly based on publication performance or social communication and interaction. This is by no means trivial especially since it is considerably easier to achieve a significant RG score by communication than with publications. This is the portal operators’ intention in order to create activity and traffic on the website. It is certainly not scientific.

This discussion reveals the difficulty with which altmetrics in general are confronted: on the one hand, it would be desirable to establish an alternative to bibliometrics, but on the other hand it is not easy to interpret the figures generated. A great many figures are obtained, especially those that are easy to generate and are usually presented as absolute values or percentages. Bibliometrics has long since developed past this stage, for example by using standardized bibliometric indicators. On the one hand, simple figures and metrics are not unproblematic (e.g. journal impact factor), but on the other hand they are necessary in order to make progress and to initiate a discussion about these metrics.

## Summary and outlook

The results of this survey were largely able to answer the original questions. The survey participants seemed to spend very little time on searches and on updating their profiles largely because ResearchGate does not seem to be the first port of call for scientific searches and since ResearchGate itself simplifies profile updates. The investigation of usage patterns revealed that questions were more frequently answered than asked although neither activity was practised by the majority of respondents. The participating scientists tend to believe that they do not experience any job-related benefits by using ResearchGate although the majority are of the opinion that they may have increased their academic influence and reputation. The social network does not seem to play a major role in the respondents’ environment since few of them experienced any social pressure from colleagues or superiors and they themselves also rarely advise others to use ResearchGate. With respect to the future, most respondents are in agreement that they do not necessarily intend to spend more time using ResearchGate. Many participants are critical of the RG score because the calculation is not obvious and the informative value of the metric is therefore also unclear. Nevertheless, many of them are of the opinion that it is important for them to have the highest possible RG score.

The survey model developed here could be taken over, adapted, or further developed in future research work in order to implement a similar survey for related academic social networks such as Mendeley or Academia.edu. Using those findings, a comparison could be made with the results of the present survey in order to create a more differentiated picture of usage patterns and scholars’ opinions on the topic of altmetrics and their sources in the form of social networks. It should be noted that ResearchGate is still not to be found among the altmetric sources in databases like Altmetric.com or PlumX despite its increasing importance in the scientific world. This is due to the restraint of the operators of ResearchGate to provide access to their data.

It remains to be noted that due to the fact that scientists tend to not fully understand the calculation of the RG-Score, the importance seems to be relatively high based on the results of the survey. This is comparable to the significance of the Impact Factor from Web of Science which is also not fully transparent.
